# Addressing Glutathione Redox Status in Clinical Samples by Two-Step Alkylation with N-ethylmaleimide Isotopologues

**DOI:** 10.3390/metabo10020071

**Published:** 2020-02-16

**Authors:** Tamara Tomin, Matthias Schittmayer, Ruth Birner-Gruenberger

**Affiliations:** 1 Institute of Chemical Technologies and Analytics, Faculty of Technical Chemistry, Vienna University of Technology—TU Wien, Getreidemarkt 9/164, 1060 Vienna, Austria; tamara.tomin@tuwien.ac.at; 2 Diagnostic and Research Institute of Pathology, Diagnostic and Research Center for Molecular BioMedicine, Medical University of Graz, Stiftingtalstrasse 6, 8010 Graz, Austria; 3 Omics Center Graz, BioTechMed-Graz, Stiftingtalstrasse 24, 8010 Graz, Austria

**Keywords:** GSH, GSSG, oxidative stress, NEM, d5-NEM

## Abstract

Determination of the ratio of reduced to oxidized glutathione is of profound clinical interest in assessing the oxidative status of tissues and body fluids. However, this ratio is not yet a routine clinical parameter due to the analytically challenging interconversion of reduced (free) glutathione to oxidized (bound) glutathione. We aimed to facilitate this ratio determination in order to aid its incorporation as a routine clinical parameter. To this end, we developed a simple derivatization route that yields different isotopologues of N-ethylmaleimide alkylated glutathione from reduced and oxidized glutathione (after its chemical reduction) for mass spectrometric analysis. A third isotopologue can be used as isotopic standard for simultaneous absolute quantification. As all isotopologues have similar chromatographic properties, matrix effects arising from different sample origins can only impact method sensitivity but not quantification accuracy. Robustness, simplified data analysis, cost effectiveness by one common standard, and highly improved mass spectrometric sensitivity by conversion of oxidized glutathione to an alkylated glutathione isotopologue are the main advantages of our approach. We present a method fully optimized for blood, plasma, serum, cell, and tissue samples. In addition, we propose production of N-ethylmaleimide customized blood collection tubes to even further facilitate the analysis in a clinical setting.

## 1. Introduction

Glutathione (γ-L-glutamyl-L-cysteinyl-glycine) is the main endogenous, thiol-based antioxidant existing in reduced (GSH) and oxidized (GSSG) forms in mammalian cells. More than 98% of the total glutathione pool consists of GSH in the concentration range of 1–10 mM, while GSSG accounts for the residual 1–% [1,2,3]. GSH can act as a nucleophilic adaptor for various different electrophiles [1]. However, its predominant role is to remove hydrogen peroxides by acting as a cofactor for the selenium-based enzyme family of glutathione peroxidases (GPx) [3], a reaction during which it oxidizes to GSSG [4]. While GSSG is recycled by glutathione reductase (GR) to GSH in an NADPH-dependent manner [5], extensive oxidative stress can alter the GSH/GSSG ratio as a consequence of either insufficient capacity of GR or redox imbalance [6]. Therefore, the GSH-to-GSSG ratio is used as a readout of tissue redox state [7].

Disturbed glutathione homeostasis has been reported in various different pathological conditions [8]. However, as the cellular GSH concentration is approximately two orders of magnitude higher than the GSSG concentration, postsampling oxidation of even a small fraction of GSH to GSSG can lead to a severe bias of the concentration measured for the latter. In order to prevent artificially high GSSG levels, it is now widely accepted that all biological samples should be treated with a thiol-quenching reagent which captures free GSH immediately after sampling and prior to further sample processing [9,10]. The most commonly used GSH conjugating reagent is N-ethylmaleimide (NEM) due to its cell permeability and fast reaction kinetics. NEM has the additional advantage of also inactivating GR, which could potentially reduce GSSG after GSH–NEM coupling and lead to an underestimation of cellular GSSG [9].

Here, we show the feasibility of labeling both reduced and oxidized glutathione (after chemical reduction) with different isotopically labeled NEM in a simple, straightforward sample preparation procedure for subsequent analysis by a targeted reversed-phase liquid chromatography coupled with tandem mass spectrometry (LC-MS/MS) method. We demonstrate that our method is approximately 10-fold more sensitive than direct GSSG analysis and show how our approach can be applied to cell culture, blood, plasma, serum, and tissue samples. 

## 2. Results

### 2.1. Method Overview

Typically, 5 µL of blood, 3–5 mg of tissue sample, or 300,000 cells were used as starting material. Therefore, the method is also suitable for samples acquired by minimally invasive techniques such as fine needle aspiration biopsies (FNAB) (~500,000 cells per FNAB [11]).

**Figure 1:**
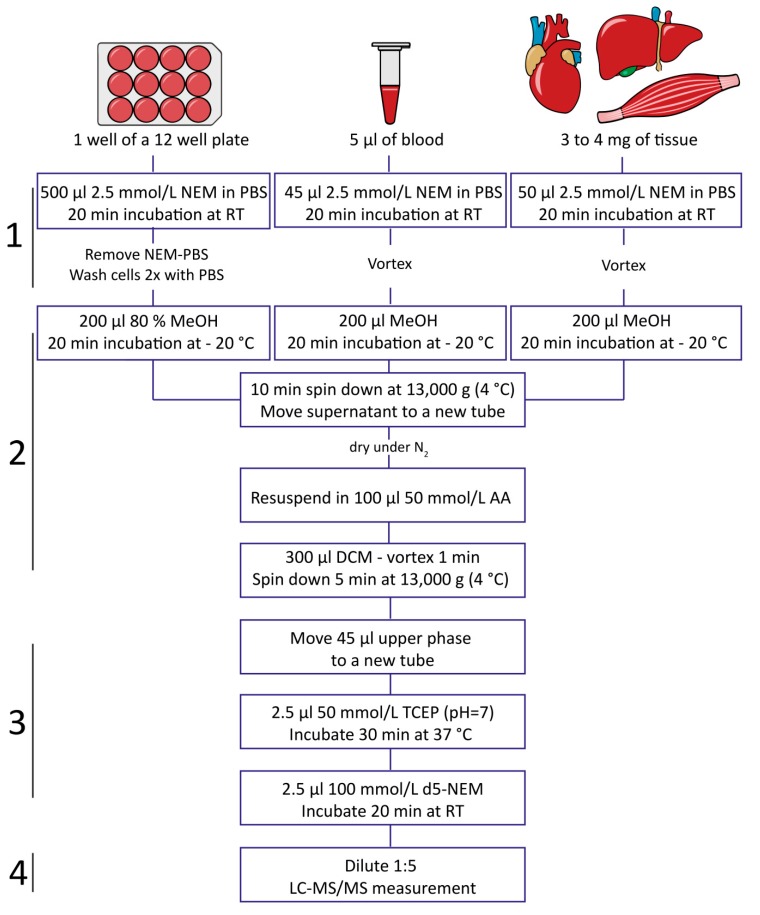
Workflow of reduced-to-oxidized-glutathione ratio determination by isotope labeling. In the first step (1), N-ethylmaleimide (NEM) was used to derivatize free glutathione (GSH) in a sample-type-specific manner. So far, the method has been optimized but is not limited to cells, blood, and tissues. In the following step (2), proteins and excess NEM were removed by methanol (MeOH) precipitation and dichloromethane (DCM) extraction. Next (3), non-protein-bound oxidized thiols in the samples (including oxidized glutathione (GSSG)) were reduced and newly formed GSH was then derivatized to GSH-d_5_-NEM. Finally, all analytes were measured simultaneously by multiple reaction monitoring (MRM)-based LC-MS/MS (4). Corresponding chemical reactions are displayed in Suppl. Figure S1. PBS: phosphate-buffered saline, RT: room temperature, AA: ammonium acetate.

The workflow involved first (Figure 1, step 1 and Suppl. Figure S1.1) the quenching of free glutathione with 2.5 mM NEM for 20 min at room temperature (RT). NEM is an ideal derivatization reagent for this purpose as it is cell permeable, reacts quickly and to completion (Figure 1, step 1 and Suppl. Figure S2), and also inactivates GR [9]. To ensure complete derivatization of more challenging samples such as tissue pieces (Suppl. Figure S2), samples were incubated for 20 min at room temperature. Complete GSH derivatization in tissue was achieved by vortexing 3–4 mg of skeletal or cardiac muscle tissue cut into small pieces in NEM containing phosphate-buffered saline (PBS), without the need for additional lysis steps, thus greatly simplifying sample handling (Suppl. Figure S2B). Cell culture samples can be either incubated in NEM-containing PBS (NEM/PBS) like tissue and blood or directly lysed in 80% methanol containing NEM, without any influence on the total reduced-to-oxidized-glutathione ratio of the cells (Suppl. Figure S3).

**Figure 2:**
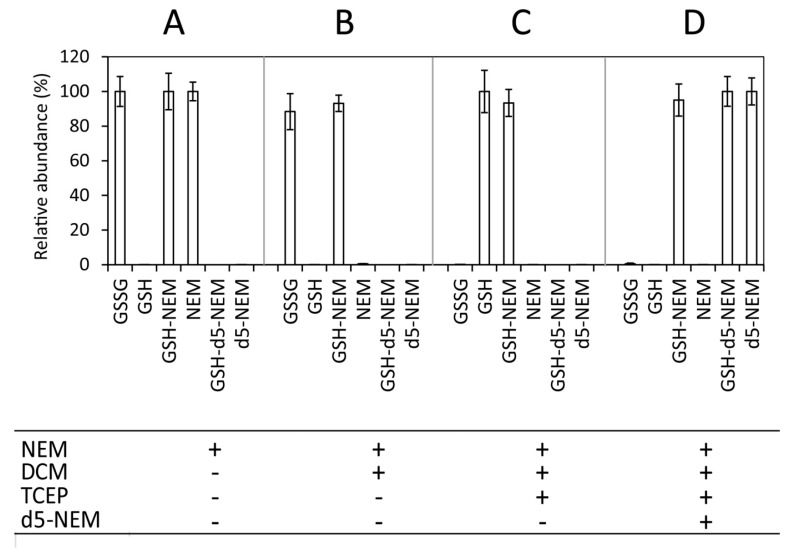
Step-by-step analysis of completeness and selectivity of derivatization of reduced-to-oxidized-glutathione ratio determination workflow. Four technical replicates of 100 µmol/L of GSH and 10 µmol/L of GSSG standard were mixed and processed as indicated by plus icons in the table below the panel (steps **A–D**). Nonderivatized samples resulted in a massive standard deviation (S.D.) for both GSH and GSSG due to oxidation; thus, they are not shown in this figure. Bars represent means of MRM signal areas normalized on the mean of MRM signal area of the corresponding analyte ± S.D. at its first appearance in the panel: all GSH–NEM, GSSG, and NEM signals were normalized on signals of GSH–NEM, GSSG, and NEM, respectively, measured after the first step of the procedure (NEM addition, panel A). Due to high variation of GSH without the addition of NEM, GSH was normalized on the GSH signal measured after GSSG reduction (panel C). GSH-d_5_-NEM and d_5_-NEM signals were normalized on the signals of GSH-d_5_-NEM and d_5_-NEM after reduction/second alkylation step (panel D).

After all free GSH was quenched, cold 80% methanol (MeOH) was used to precipitate and remove proteins from the sample (Figure 1, step 2). At this step, the internal standard (IS) can be introduced to monitor extraction efficiency and analyte recovery of the subsequent extraction steps. Following centrifugation, the protein-free supernatant was transferred to a new reaction tube and dried under a stream of nitrogen. As NEM alkylation is best performed at near neutral pH for optimal selectivity and reactivity [12], dried samples were resuspended in 100 µL of 50 mM ammonium acetate (AA) (pH = 7) and excess NEM was removed by a single extraction with dichloromethane (DCM) (sample:DCM v:v 1:3), resulting in removal of NEM beyond the limit of quantification (Figure 2B). Any residual NEM was additionally quenched in the next step. Upon DCM extraction, the aqueous phase was transferred to a new tube and subjected to reduction with Tris(2-carboxyethyl)phosphine (TCEP), followed by d_5_-NEM derivatization of newly released GSH (Figure 1, step 3 and Suppl. Figure S1.2). TCEP is a widely employed reducing agent in protein and peptide chemistry [13]. It is less well known that TCEP is also highly reactive towards maleimides [14] (Suppl. Figure S4). TCEP (added to 2.5 mM final concentration; stock solution buffered to pH = 7), therefore, fulfilled a double role by scavenging residual nonisotopically labeled NEM and also quantitatively reducing GSSG (Figure 2C).

**Figure 3:**
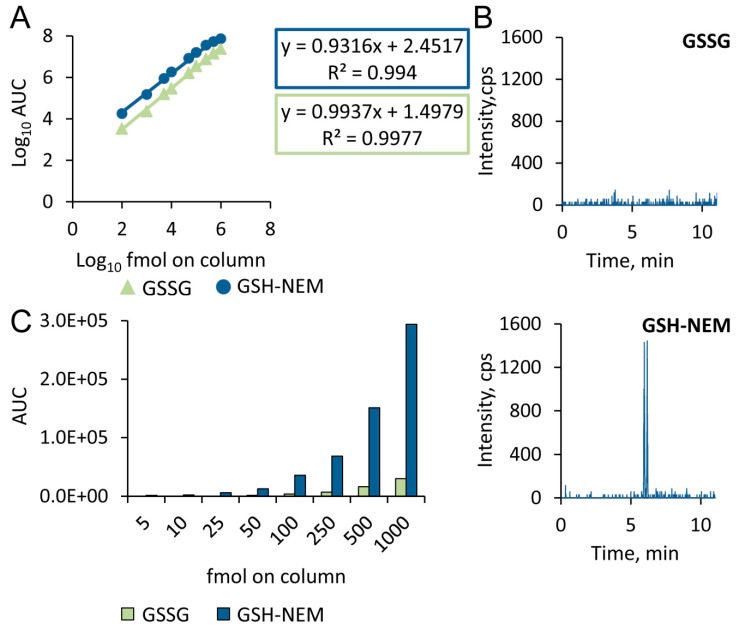
GSH–NEM is a superior analyte to GSSG in terms of sensitivity. (**A**) Calibration curves of GSH–NEM (y = 0.932x + 3.38, R2 = 0.994) and GSSG (y = 0.994x + 2.49, R2 = 0.9977). (**B**) 25 fmol on column of GSSG was not directly detectable (top), but its reduction and NEMylation resulted in readily quantifiable GSH–NEM twin peaks (bottom, see methods section). (**C**) Comparison of signal area responses in the low concentration range (5–1000 fmol on column) when GSSG was measured directly as compared to in the form of GSH–NEM.

To rule out the labeling of newly formed GSH with residual nonisotopically labeled NEM, we performed a control experiment using a mixture of GSH and GSSG standards in the concentration ratio of 10:1 (100 µM GSH and 10 µM GSSG). While this ratio is far below the naturally occurring ratios, the GSH content is in the concentration range of a biological sample after dilution for LC-MS measurement. Addition of NEM to a final concentration of 2.5 mM alkylated all free GSH (Figure 2A), and even under these extreme conditions of high GSSG favoring off-target labeling of GSH derived from GSSG, no significant contribution to the nonisotopically labeled GSH–NEM pool was observed (Figure 2B,C). Finally, an excess of d_5_-NEM (5 mM) was added to quantitatively label GSSG-derived GSH (Figure 2D and Suppl. Figure S1.3). Remarkably, GSSG reduction following d_5_-NEM labeling of released GSH resulted in a 10-fold sensitivity increase (compared with measuring GSSG alone), as described in detail in the next section (Figure 3).

Lastly, all three analytes (GSH–NEM, GSH-d_5_-NEM, and ^13^C_2_, ^15^N-GSH-d_5_-NEM (IS)) were then simultaneously measured by LC-MS/MS using a multiple reaction monitoring (MRM)-based approach (Figure 1, step 4).

The method was tested and optimized for cell culture samples exposed to oxidative stress (see supplement (Supplemental methods and results, Suppl. Figures S3 and S5)); tissues samples (Supplemental methods, Suppl. Figure S2); as well as blood, serum, and plasma. As blood and blood derivatives are the most frequently collected clinical samples, we dedicated special attention to blood sample collection and preparation for glutathione analysis.

### 2.2. Sensitivity of the Approach

The signal response of GSH–NEM was similar to GSH but about 5-fold higher than that of GSSG, while noise levels were comparable throughout the whole dynamic range (Figure 3A, Suppl. Figure S6). As GSSG reduction results in two molecules of GSH, overall sensitivity of the method for GSSG detection after derivatization to GSH-d_5_-NEM was roughly 10-fold better than direct GSSG measurement (Figure 3C).

The boost in sensitivity was also reflected by the lower detection limits for GSSG. For example, 25 fmol of GSSG on column was below the limit of detection (LOD, monitored as MRM trace of GSSG, RT = 3.71 min), while the same GSSG amount after reduction and alkylation with NEM resulted in a reliably quantifiable GSH–NEM peak with a signal-to-noise ratio (S/N) of more than 10 (Figure 3B). Tenfold greater sensitivity (Figure 3C) yielded a lower LOD and limit of quantification (LOQ) for GSH–NEM. LOD for GSSG was 50 fmol on column when measured directly as compared with 5 fmol on column when measured as GSH–NEM (with S/N ratios of 7.5 and 7.0, respectively). LOQ was 100 fmol on column for GSSG (S/N = 11.5) and 10 fmol on column for GSSG measured as GSH–NEM (S/N = 13), respectively.

### 2.3. Impact of Sample Processing on GSH/GSSG Determination in Blood

The GSH/GSSG ratio in blood has been reported to be 3–20-fold higher as compared with solid tissues and cultured cells [15]. In red blood cells (RBCs), GSH accounts for ~99.8%, GSSG for ~0.1%, and S-glutathionylated hemoglobin for 0.05% of total glutathione, meaning that all the glutathione in RBCs is almost exclusively represented as GSH [16]. Therefore, when GSH and GSSG levels in blood are assessed, even the slightest artificial oxidation of GSH during sample preparation can lead to an overestimation of GSSG even as high as 7–50-fold [17]. To monitor this effect, we investigated how delayed NEM addition affects detected GSH and GSSG levels. Blood vacuum containers for full blood, plasma, and serum were customized by adding either NEM or PBS (control tubes) prior to being employed for collecting blood from five healthy volunteers. Plasma and serum were prepared according to the Early Detection Research Network SOP [18]. In brief, EDTA plasma was centrifuged at room temperature immediately after collection for 10 min. Serum was allowed to clot at room temperature for 30 min in an upright position before being centrifuged for 20 min. NEM was added to both plasma and serum control tubes (which had been customized with PBS only) immediately after centrifugation. Whole-blood samples were stored at 4 °C for 30 min prior to addition of NEM to the control tubes.

**Figure 4:**
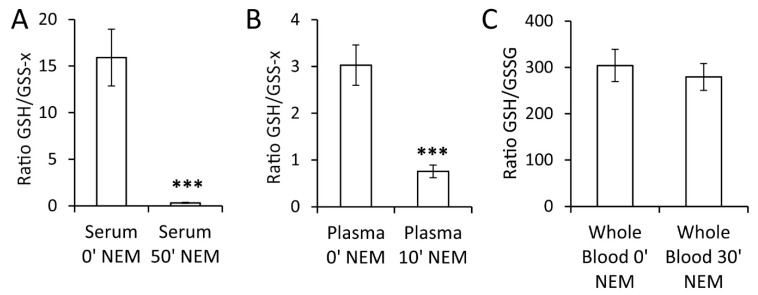
Delay in NEM addition resulted in lower GSH/GSSG ratios in serum and plasma samples. (**A**) GSH/GSS-x ratio (with “x” being any soluble thiol) was reduced in serum if NEM was added after preparation (50 min at room temperature (RT)) instead of immediately. (**B**) Effect of plasma preparation time (10 min at RT) prior to NEM addition on GSH/GSS-x ratio of plasma. (**C**) Thirty minutes of incubation of whole blood at 4 °C did not alter the GSH/GSSG ratio. Bars represent mean ratio ± error propagated S.E.M. (n = 5, Student *t* test, *** *p*-value < 0.001).

In contrast to RBCs, where GSH is the most abundant thiol, free cysteine is reportedly the most abundant thiol in plasma [16,19,20]. As the oxidation of thiols in cell-free blood preparations is not an enzymatic but a chemical process, GSH (released from blood cells) will inevitably also form mixed disulfides with other highly abundant thiols such as cysteine [20,21]. Therefore, when applying our proposed protocol, which involved reduction before a second alkylation step, especially in serum and plasma, we were not measuring only GSSG but all oxidized, non-protein-bound glutathione (labeled as GSS-x in Figure 4). Potential benefits of such an approach are further addressed in the discussion section.

Delays between sample collection and NEM addition resulted in a reduction of measured reduced-to-oxidized-glutathione ratios in serum and plasma but not in whole blood (Figure 4). As serum preparation was the lengthiest procedure (50 min at RT), the effect was more pronounced for serum (Figure 4A) than for plasma (10 min at RT) (Figure 4B). It should be noted that in serum and plasma, absolute GSH concentrations represented only 0.1–3% of those measured in full blood (Suppl. Table S1). Delayed addition of NEM is especially of concern when strictly time-controlled processing cannot be guaranteed, as is often the case in a clinical environment. Immediate derivatization of GSH is therefore inevitable for accurate determination of the redox state. If instantaneous NEM addition is not possible, whole-blood samples can still be analyzed after being kept refrigerated at 4 °C for up to 30 min without a detectable effect on the GSH/GSSG ratio (Figure 4C).

While it has been proposed that addition of NEM in higher concentrations to blood tubes before plasma preparation can potentially lead to leakage of GSH–NEM from the RBCs [10], our final NEM concentration (2.5 mM) was well below the range where leakage was observed.

**Figure 5:**
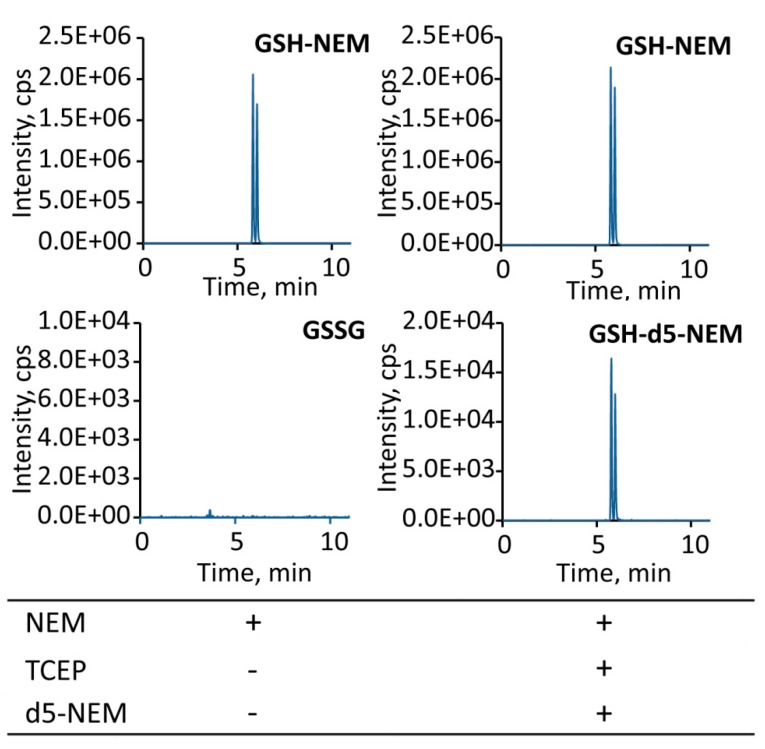
Detection of GSSG in minimal amounts of whole blood. In 1 µL of whole blood, no quantifiable GSSG peak was directly detectable (left), while reduction of GSSG with Tris(2-carboxyethyl)phosphine (TCEP) to GSH and subsequent alkylation with d5-NEM resulted in clearly detectable GSH-d_5_-NEM (right).

The improved sensitivity for quantification of oxidized glutathione using our approach was apparent when analyzing GSSG from 1 µL of whole blood (Figure 5). While direct measurement of GSSG did not yield a quantifiable MRM peak, reduction with TCEP and subsequent alkylation with d_5_-NEM resulted in a prominent GSH-d_5_-NEM peak (Figure 5).

## 3. Discussion

The biological function of glutathione has been intensively investigated since its discovery in the 1920s [22]. Traditional colorimetric assays for determination of GSH are based on the reaction of the free thiol group with Ellman’s reagent (5,5′-dithiobis-2-nitrobenzoic acid, DTNB) or a similar dye. The obvious drawback of this approach is that Ellman’s reagent reacts with any soluble thiol and other small polar thiol-containing molecules, for example, cysteine (for an excellent overview of soluble thiols, see Sutton et al. [10]), adding to the observed signal. For colorimetric detection of GSSG, the oxidized form is usually reduced either chemically or enzymatically. The latter approach has the advantage of substrate specificity of the enzyme; however, it is tedious to perform and is easily influenced by different experimental factors [15]. In addition to colorimetric assays, there are many other methods available for glutathione measurements, spanning from fluorometric, electrochemical, and high-performance liquid chromatography coupled with ultraviolet detection (HPLC-UV) to capillary electrophoresis and others [23]. With the rise of LC-MS/MS, the requirement of enzyme coupling for selective GSSG detection has diminished. However, the need to protect free GSH from oxidation remains. Therefore, blocking free GSH with alkylating reagents is still widely employed in the field [9,16,24,25], with the low abundance of GSSG limiting method sensitivity. Moreover, accurate quantification and determination of the GSH/GSSG ratio require a careful compensation of matrix effects with expensive isotopically labeled standards [24], as matrix effects differ depending on the sample source, be it cell culture, different tissues, or body fluids. Recent considerable efforts provide main guidelines for sample collection and processing, especially when it comes to sample handling and avoiding artefacts [10,15]. Overall, the most likely limiting factor for determination of reduced and oxidized glutathione is the less abundant oxidized counterpart. While some of the fluorescent methods report similar sensitivity as ours [23], other assays tend to require separate sample preparation and/or tedious procedures for GSH and GSSG measurements, which might render them less practical. In addition, direct measurement of GSH and GSSG using LC-MS/MS in most cases requires two separate heavy-isotope-labeled internal standards, which can add to the cost of the individual analysis.

In this study, we demonstrated how GSH and GSSG-derived GSH can be individually measured as isotope-labeled GSH–NEM derivatives in a single LC-MS/MS experiment. Sample processing was carried out in a simple straightforward procedure, minimizing sources of error and analyte loss. The isotopologue-based design of the method largely compensates for different matrix effects, as all analytes coelute from liquid chromatography and are ionized simultaneously. Another major advantage of the method is increased sensitivity. Determination of GSSG after its derivatization to GSH-d_5_-NEM is at least 10 times more sensitive than direct measurement of GSSG. Therefore, only tiny sample amounts are required, thus rendering the method compatible with minimally invasive clinical sampling procedures.

Blood is probably the most frequently processed form of a clinical sample. We investigated how the delay caused by whole-blood storage or standardized serum and plasma preparation procedures affects the detected reduced-to-oxidized-glutathione ratio. Interestingly, the whole-blood GSH/GSSG ratio was not significantly altered after 30 min at 4 °C. As the bulk of GSH and GSSG are localized within intact, metabolically active blood cells, GSH is not exposed to an oxidizing environment and freshly formed GSSG is presumably constantly recycled to GSH by GR in an NADPH-dependent manner as long as nutrients are available. In contrast, reduced to oxidized glutathione in cell-free blood preparations is severely biased by the time-dependent oxidation of GSH due to its exposure to air. Surprisingly, only a small part (Suppl. Table S1, ~5% in serum) of the oxidized GSH is actually recovered after reduction and derivatization as heavy labeled GSH–NEM. One potential explanation for this is unselective formation of mixed disulfides of GSH with proteins in the extracellular environment upon exposure to air. This way, a large fraction of GSH would be removed at the protein precipitation step. However, free GSH detected in serum is also a factor of six higher than in plasma. The most likely reason for this is increased hemolysis during blood coagulation, which requires incubation for 30 min followed by 20 min centrifugation, both at room temperature. Continuous release of intracellular GSH–NEM and GSSG, with its far higher GSH/GSSG ratio than observed in plasma, could very well explain both the intermediate GSH/GSSG ratio observed in serum as compared with whole blood and plasma as well as the increased GSH–NEM concentration in serum as compared with plasma.

It is clear that with the proposed reduction and second alkylation approach we are not only measuring GSH released from GSSG but also all the other non-protein-bound forms of oxidized glutathione (since proteins are removed prior to the reduction step). Contribution of mixed disulfides in cellular systems seems to be insignificant, as the GSH/GSSG ratio is, as mentioned, under tight enzymatic control. This is nicely corroborated by the fact that our measured absolute GSSG values of whole blood match previously published reports [17,26]. However, in cell-free systems, such as plasma or serum, oxidation of GSH is a chemical process and here, using GSSG as the only readout of glutathione oxidation, might actually lead to underestimation of the extent of glutathione oxidation. With higher contents of cysteine and homocysteine in plasma and therefore higher probability of formation of mixed GSH–cysteine and GSH–homocysteine disulfides [20,21], it can be argued that our approach can provide better assessment of the overall glutathione oxidation state. We propose to use the ratio of GSH/GSS-x (with “x” being any soluble thiol) instead of GSH/GSSG as a more meaningful readout of chemical oxidation in the case of cell-free systems. Therefore, as compared with other reports that found GSSG concentrations in the mid-nanomolar range [10,19], we report low micromolar values of oxidized glutathione (Suppl. Table S1). Altogether, we propose a convenient and robust procedure which can be easily implemented and used in various clinical workflows, especially for the analysis of oxidized glutathione in cell-free blood preparations such as serum and plasma.

## 4. Materials and Methods

For a detailed method description of standards, cell culture samples, and mass spectrometry, refer to the supplement. If not stated otherwise, all chemicals were purchased from Sigma-Aldrich (St. Louis, MO, USA).

### 4.1. Standards

Standards for GSH and GSSG were prepared as 10 mM stock solutions in ultrapure water. For calibration curves, stock solutions were diluted in 50 mM AA buffer (pH = 7.0). NEM and d_5_-NEM were prepared as 100 mM stock solutions in water. TCEP was used as 50 mM stock solution prepared in 50 mM AA buffered to pH = 7.0. GSH stock solution for calibration curves was prepared in ultrapure water with addition of TCEP to a final concentration of 10 mM. The stock solution of the IS (^13^C_2_,^15^N-GSH-d_5_-NEM) was prepared by reacting 2 mM glutathione-(^13^C_2_,^15^N) (Cambridge Isotope laboratories, MA, USA) with 50 mM d_5_-NEM in 50 mM AA, with subsequent extraction of residual d_5_-NEM with DCM. Final IS concentration was determined by analyzing a 1:100 dilution in 50 mM AA by LC-MS/MS and matching signal intensity to the calibration curve of unlabeled GSH–NEM.

### 4.2. Liquid Chromatography Coupled with Tandem Mass Spectrometry

Chromatography was carried out on a Dionex UltiMate 3000 system equipped with a Zorbax SB-C18 column (50 × 4.6 mm, 1.8 µm, Agilent, Santa Clara, CA, USA). The following gradient employing solvent A (0.1% formic acid in water) and solvent B (0.1% formic acid in acetonitrile) at a flow rate of 0.3 mL/min was applied: 0–10 min, 1–30% B; 10–15 min, 30–70% B; 15–20 min, 1% B. Injection volume was 10 µL. The ABSciex 4000 QTRAP mass spectrometer used for detection was operated in positive MRM mode. A list of transitions with retention times and collision energies (CEs) of analytes is shown in Table 1. Global instrument parameters: curtain gas: 20 (arbitrary units); collision gas: high; ion spray voltage: 4500 V; temperature: 450 °C; ion source gas: 1 and 2: 25 (arbitrary units) and 40 (arbitrary units); declustering potential: 50 V; and entrance potential: 10 V. As GSH–NEM is a diastereomer [9,25], all three GSH–NEM analytes (GSH–NEM, GSH-d_5_-NEM, and ^13^C_2_, ^15^N-GSH-d_5_-NEM) elute as twin peaks. The sum of areas under both peaks was used for integration and quantification. We also detected two peaks for the transition for NEM, but only the peak with the higher retention time showed the expected UV absorbance at 305 nm, suggesting that the other peak may resemble a hydrolysis product of NEM (Suppl. Figure S7).

### 4.3. Blood, Plasma, and Serum Sample Collection and Processing

Blood samples were collected from healthy volunteers (3 male, 2 female; mean age 36 ± 3 years) in VACUETTE^®^ 3.5 mL tubes with Z Serum Separator Clot Activator 13 × 75 red cap/yellow ring, nonridged (Greiner bio-one, Rainbach im Mühlkreis, Austria) or in VACUETTE^®^ 3.5 mL K2 EDTA tubes, pull cap, lavender (Greiner bio-one, Austria). All blood collection tubes were customized prior to blood sampling as follows: To each tube for direct NEMylation, 100 µL of 87.5 mM NEM in PBS was added to reach a final concentration of 2.5 mM NEM after blood would fill the tube (NEM 0 min samples). To each tube of control samples (delayed NEM addition samples), 100 µL of PBS was added instead. For serum collection, all blood samples were coagulated for 30 min at RT. After coagulation, samples were centrifuged at 1300× *g* at RT for 20 min. All plasma samples were immediately centrifuged for 10 min at RT at 1300× *g*. Whole-blood samples were kept at 4 °C for 30 min. After these dedicated processing times and temperatures (50 min at RT for serum, 10 min at RT for plasma, and 30 min at 4 °C for whole blood), 5 µL of each sample was pipetted out of the VACUETTE^®^ tubes into 1.5 mL micro centrifuge tubes containing either 45 µL PBS (NEM 0 samples) or 45 µL of 2.5 mM NEM in PBS (delayed NEM addition samples). Further sample processing was carried out as shown in Figure 1.

The use of human biomaterials was approved by the Ethics Committee of the Medical University of Graz (26-282 ex 13/14) and conformed with all pertaining regulations and the principles of the Declaration of Helsinki [27].

## 5. Statistics

If not stated otherwise, data are reported as mean values ± standard deviation (S.D.). For significance testing, an unpaired Student’s *t* test was performed with a *p*-value of 0.05 as the significance threshold.

## Figures and Tables

**Table 1:** MRM transitions and parameters per analyte. For each compound, transition 1 (marked in bold) was used for quantification. Dwell time was 35 ms. CE: collision energy; CXP: collision cell exit potential; IS: internal standard.

Analyte Name	Q1 Mass (amu)	Q3 Mass (amu)	CE (V)	CXP (V)
**GSSG** (*Transition 1*)	613.2	355.3	33	14
GSSG (*Transition 2*)	613.2	484.2	25	12
**GSH** (*Transition 1*)	308	179	19	15
GSH (*Transition 2*)	308	76.23	47	15
**GSH-NEM** (*Transition 1*)	433	201	35	15
GSH-NEM (*Transition 2*)	433	84.2	59	15
**GSH-d_5_-NEM** (*Transition 1*)	438	206	35	15
GSH-d**_5_**-NEM (*Transition 2*)	438	84.2	59	15
**NEM** (*Transition 1*)	126	80	25	12
NEM (*Transition 2*)	126	98	19	16
**d_5_-NEM** (*Transition 1*)	131	80	25	12
d**_5_**-NEM (*Transition 2*)	131	98	19	16
**^13^C_2_,^15^N-GSH-d_5_-NEM** (**IS**)	441	206	35	15

## Supplementary Materials

The following are available online at https://www.mdpi.com/2218-1989/10/2/71/s1, Supplemental Material containing Supplementary methods: Cell culture and processing, Human tissue specimens and processing, Supplementary results: Analysis of cells subjected to oxidative stress, Table S1: Absolute GSH and GSSG concentrations in serum, plasma and whole blood of healthy volunteers, Figure S1: Chemical reactions involved in the procedure, Figure S2: Completeness of derivatization of GSH in tissue samples, Figure S3: Cell culture samples can be either incubated in NEM/PBS or harvested directly in NEM/MeOH without affecting GSH/GSSG ratio, Figure S4: TCEP reacts with d_5_-NEM yielding a product of the mass to charge ratio (m/z) of 382, Figure S5: Cellular oxidative stress reflected by reduced GSH/GSSG ratio, Figure S6: Similar signal response of GSH and GSH-NEM, Figure S7: NEM MRM trace results in two peaks but only one corresponds to intact NEM.
