# Supplementary material for: Addressing Glutathione Redox Status in Clinical Samples by Two-Step Alkylation with N-ethylmaleimide Isotopologues
Source: Metabolites. 2020 Feb 16;10(2):71. doi: 10.3390/metabo10020071 (PMC7074022; doi:10.3390/metabo10020071)
Supplement: Supplementary file 1 [file metabolites-10-00071-s001.pdf]

SUPPLEMENTAL MATERIAL

Addressing glutathione redox status in clinical samples by two-step alkylation with N-ethylmaleimide isotopologues

Tamara Tomin[*1,2,3], Matthias Schittmayer[*$1,2,3], Ruth Birner-Gruenberger[$1,2,3]

[1] Institute of Chemical Technologies and Analytics, Faculty of Technical Chemistry, Vienna University of Technology - TU Wien, Getreidemarkt 9/164, 1060 Vienna, Austria

[2] Diagnostic and Research Institute of Pathology, Medical University of Graz, Stiftingtalstrasse 6, 8010 Graz, Austria

[3] Omics Center Graz, BioTechMed-Graz, Stiftingtalstrasse 24, 8010 Graz, Austria

* Contributed equally and should thus be considered as co-first authors

[$] Corresponding authors: Ruth Birner-Gruenberger and Matthias Schittmayer, Institute of Chemical Technologies and Analytics, Faculty of Technical Chemistry, Vienna University of Technology - TU Wien, Getreidemarkt 9/164, 1060 Vienna, Austria, emails: ruth.birner-gruenberger@tuwien.ac.at and matthias.schittmayer@tuwien.ac.at

SUPPLEMENTARY METHODS

Cell culture and processing

A549 and HCC827 human lung carcinoma cells were obtained from CLS Cell Lines Service, Eppelheim, Germany, and were cultured in RPMI medium supplemented with 10 % fetal bovine serum and 2 mM glutamine in a 5 % CO_2, 37 ˚C incubator. For H_2O_2 treatment, cells were seeded in 12-well plates (300,000 cells/well) and after a day in culture treated with 500 µl of control (Ca^{2+}, Mg^{2+}-PBS) or 100 µM H_2O_2 in Ca^{2+}, Mg^{2+}-PBS for 15 min followed by two PBS washing steps and the NEM (2.5 mM) incubation step. The detailed workflow is depicted in Fig. 1. For differential alkylation approach cells were either incubated 20 min on RT in 500 µl 2.5 NEM in PBS and then harvested in 80% methanol or harvested directly in 80% methanol containing 2.5 mM NEM.

Human tissue specimens and processing

Skeletal and heart tissue samples were obtained from autopsy from patients with no known skeletal or heart muscle disease. Tissue (4-7mg) was processed according to the workflow shown in Fig. 1. For tissue lysis 4 x 10 s sonication steps at 70 % amplitude were performed (Bandelin, Germany). The use of human biomaterials was approved by the Ethics committee of the Medical University of Graz (26-282 ex 13/14) and conformed with all pertaining regulations and the principles of the Declaration of Helsinki [26].

SUPPLEMENTARY RESULTS

Analysis of cells subjected to oxidative stress

A single well of a 12-well cell culture plate (around 300,000 cells) was sufficient for analysis of GSH/GSSG by our method. Even at further sample dilutions (1:5), high quality signals for all analytes were obtained. To functionally validate the procedure, we induced oxidative stress in A549 lung carcinoma cells by treatment with 100 µM H_2O_2 for 15 min. Incubation with H_2O_2 reduced GSH/GSSG ratio drastically (Suppl. Fig. S4).

SUPPLEMENTARY TABLES

Supplementary Table S1. **Absolute GSH and GSSG concentrations in serum, plasma and whole blood of healthy volunteers**. n = 5, mean values ± SEM.

	**c (GSH) [µmol/L]**	**c (GSSG) [µmol/L]**
Serum		
0 min NEM	38 ± 5	2.7 ± 0.5
50 min NEM	1.4 ± 0.2	4.3 ± 0.5
Plasma		
0 min NEM	5.9 ± 0.8	3.0 ± 0.0
10 min NEM	2.0 ± 0.3	2.6 ± 0.3
Whole blood		
0 min NEM	1249 ± 97	4.1 ± 0.3
30 min NEM	1254 ± 107	4.5 ± 0.3

SUPPLEMENTARY FIGURES

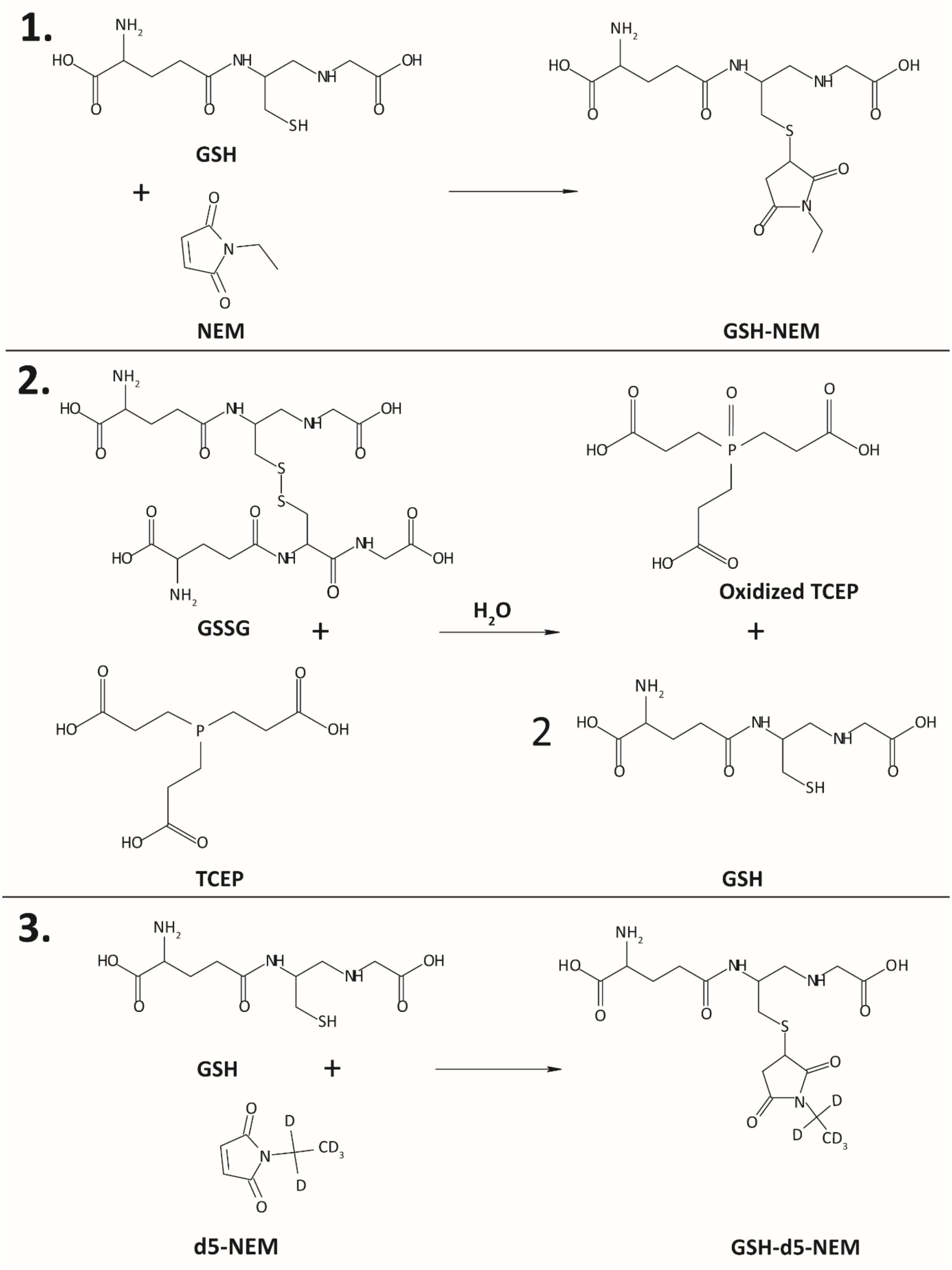

Supplementary Figure S1. Chemical reactions involved in the procedure. 1. Glutathione (GSH) reacts with N-ethylmaleimide (NEM) forming GSH-NEM; 2. Tris(2-carboxyethyl)phosphine (TCEP) quantitatively reduces oxidized glutathione (GSSG or GSS-x, GSSG shown in the figure for clarity) to two molecules of GSH, oxidizing itself in the process; 3. GSH derived from chemical reduction of oxidized glutathione is alkylated with heavy labelled (d5) NEM.

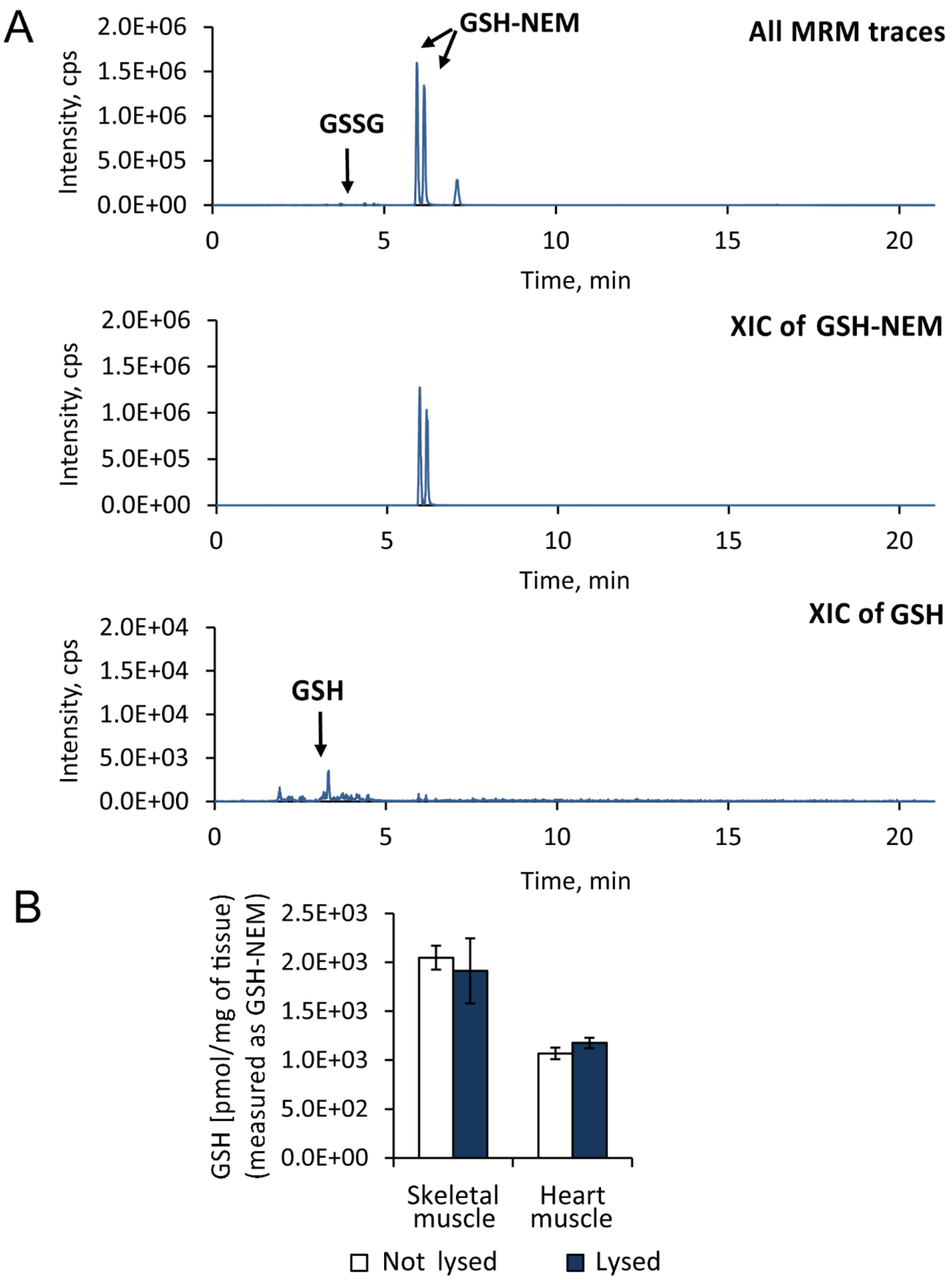

Supplementary Figure S2. Completeness of derivatization of GSH in tissue samples. A. Heart tissue sample after incubation with NEM. Upper panel shows all measured MRM traces in the sample, middle panel extracted ion chromatogram (XIC) of GSH-NEM signal while the lower one displays XIC of GSH demonstrating that upon incubation of tissue in 2.5 mM NEM no residual GSH peak detected at expected retention time of 3.2 min, only noise (please note the factor 100 difference of the intensity scale). B. Additional lysis/tissue rupture steps do not improve GSH alkylation in tissue samples (n = 3).

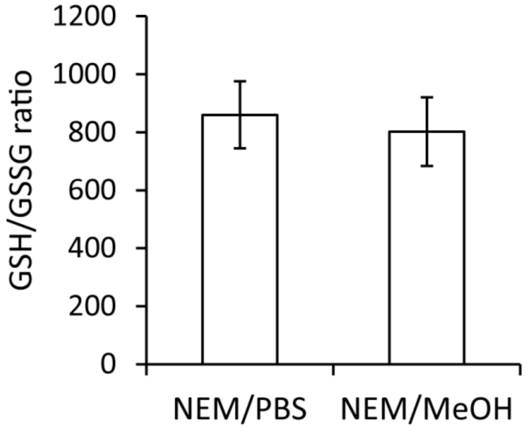

Supplementary Figure S3. Cell culture samples can be either incubated in NEM/PBS or harvested directly in NEM/MeOH without affecting GSH/GSSG ratio. HCC827 lung cancer cells were either incubated 20 min on RT in 2.5 mM NEM/PBS (NEM/PBS) then lysed with 80% methanol or lysed directly with 2.5 mM NEM in 80% methanol (NEM/MeOH). The used lysis/alkylation methods did not influence the GSH/GSSG ratio of the cells (n=5-6 samples per condition)

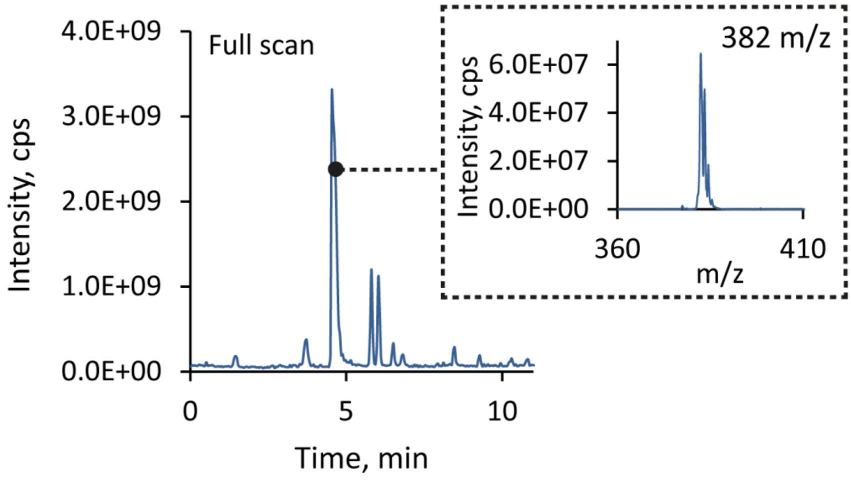

Supplementary Figure S4. TCEP reacts with d5-NEM yielding a product of the mass to charge ratio (m/z) of 382. 100 µM GSH and 10 µM of GSSG mixture was alkylated with NEM for 20 mins and then incubated for 20 min with different TCEP concentrations after which excess of d5-NEM was added. Represented is a full scan of the sample where 2 mM TCEP and 4.5 mM d5-NEM were used.

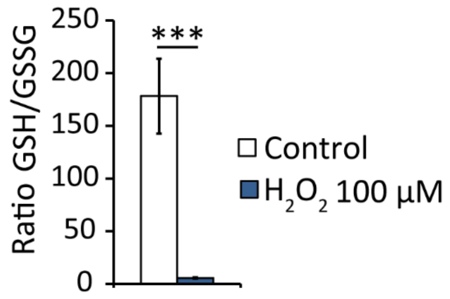

Supplementary Figure S5. **Cellular oxidative stress reflected by reduced GSH/GSSG ratio.** Cells treated with 100 µM H_2O_2 for 15 min show a vast reduction of the GSH/GSSG ratio (data from 2 independent experiments, n = 6-8 replicates per condition, data: mean ± S.E.M, Student t-test, *** p-value < 0.001).

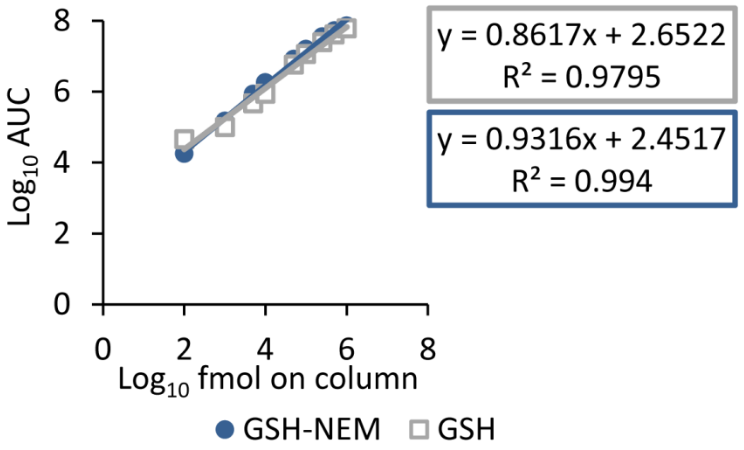

Supplementary Figure S6. Similar signal response of GSH and GSH-NEM. GSH and GSH-NEM calibration regression lines show a similar signal as GSH elutes as a wider single peak while GSH-NEM elutes as sharp double peaks from a C18 column (AUC: area under the curve in arbitrary units) with similar amounts of standards.

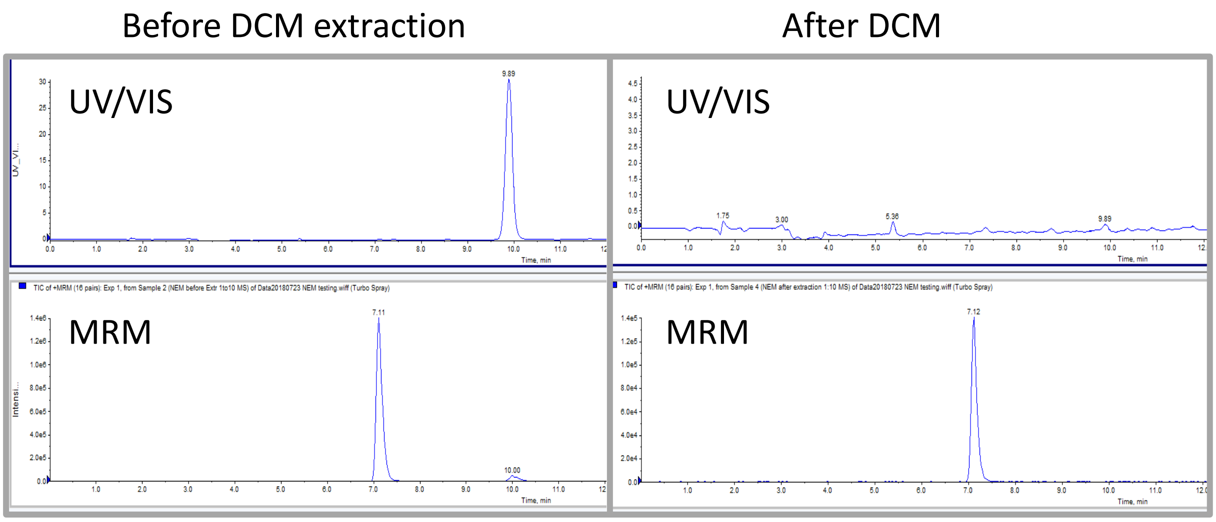

Supplementary Figure S7. NEM MRM trace results in two peaks but only one corresponds to intact NEM. Prior to DCM extraction, MRM traces for NEM appear as two separate peaks, one with retention time at 7 min and the other at 10 min. However, UV/VIS signal at 305 nm shows that only the later one resembles NEM (left panel). After DCM extraction, there is no residual signal at 305 nm yet the MRM transition peak at 7 min remains and most likely corresponds to a hydrolysis product of NEM (right panel).
